# An octa-band planar monopole antenna for portable communication devices

**DOI:** 10.1038/s41598-021-94753-w

**Published:** 2021-07-27

**Authors:** Rezaul Azim, Touhidul Alam, Md Sharif Mia, Ali F. Almutairi, Mohammad Tariqul Islam

**Affiliations:** 1 Department of Physics, University of Chittagong, Chattogram, 4331 Bangladesh; 2 Space Science Centre (ANGKASA), Institute of Climate Change (IPI), Universiti Kebangsaan Malaysia (UKM), 43600 Bangi, Malaysia; 3 Electrical Engineering Department, College of Engineering and Petroleum, Kuwait University, 13060 Safat, Kuwait; 4 Department of Electrical, Electronic and Systems Engineering, Faculty of Engineering and Built Environment, Universiti Kebangsaan Malaysia (UKM), 43600 Bangi, Malaysia

**Keywords:** Energy science and technology, Engineering

## Abstract

Due to the rapid development of wireless communication systems, good numbers of services and devices use different frequency bands and protocols. To concurrently cover all these services, the antenna in communication devices should operate over multiple frequency bands. The use of wide and multi-band antennas not only reduces the number of antennas necessary to cover multiple frequency bands but also lessens the system complexity, size, and costs. To operate over eight frequency bands to cover sixteen well-established narrow service bands, a planar monopole antenna is proposed for portable communication devices. The proposed antenna is comprised of an inverted F-shaped monopole patch with a rotated L-shaped strip and an F-shaped ground strip with a rotated L-shaped branch. The studied antenna can excite at multiple resonant modes which helps it to achieve eight measured operating bands of 789–921 MHz, 1367–1651 MHz, 1995–2360 MHz, 2968–3374 MHz, 3546–3707, 4091–4405 MHz, 4519–5062 MHz and 5355–6000 MHz. The achieved measured operating bands can cover sixteen popular narrow service bands for 4G/3G/2G, MWT, WiFi, WiMAX, WLAN, and sub-6 GHz 5G wireless communication system. The studied antenna achieved good gain, efficiency and exhibits stable radiation characteristics. Moreover, the antenna does not use any lumped element and left ample space for other circuitries which makes it easier to use in portable devices such as tablets, laptops, etc. with low manufacturing cost.

## Introduction

Currently, large numbers of wireless communication services/devices with different operating frequencies and protocols have been deployed worldwide. To simultaneously cover all these services, the antenna in portable communication devices should operate over multiple frequency bands. The use of a multi-band antenna not only lessens the number of antennas necessary to cover numbers of frequency bands but also lessens the system complexity, overall device size, and costs. An antenna in portable communication devices such as in mobile phone, a tablet should cover not only the existing frequency bands for 2G, 3G, and 4G systems, such as GSM850 (824–894 MHz), GSM900 (880–960 MHz), UMTS (1920–2170 MHz), LTE850 (869–894 MHz), LTE2190/2000S band (2180–2200 MHz), LTE3500 (3400–3600 MHz), LTE 3700 (3600–3800 MHz) and LTE5537.5 (5150–5925 MHz) but also 3.5 GHz (3.5–3.7 GHz) and 4.8 GHz (4.6–5.0 GHz) bands for newly deployed sub-6 GHz 5G system. At the same time, the popular narrowband services including 900 MHz (IEEE 802.11ah) and 5.8 GHz (IEEE 802.11ac) bands for WiFi, 3.5 GHz (3.4–3.65 GHz), and 5.5 GHz (5.25–5.85 GHz) bands for WiMAX, 5.8 GHz (5.725–5.875 GHz) band for WLAN and 1.41–1.45 GHz band for wireless medical telemetry (WMT) should be covered. As the space for the antennas in portable devices is limited, the designing of a multi-band antenna with a small size is not only a crucial requirement but also a challenging task.

In recent times good numbers of wideband and multi-band antennas have been reported^1–18^. For example, in Ref.^3^, a half-loop frame antenna for the LTE metal-casing tablet device was reported that consists of a metal casing and a rectangular-ring metal frame. With an overall volumetric size of 150.8 × 200.8 × 7 mm^3^, the presented antenna achieved dual operating bands of 746–960 MHz and 1710–2690 MHz. In Ref.^6^, a hepta-band loop antenna was presented for LTE/WWAN applications. The designed antenna has an overall dimension of 76 × 150 × 6 mm^3^ and can operate over 824–960 MHz and 1710–2690 MHz, bands. For metal-frame smartphones, in Ref. 9 a hepta-band antenna with a size of 70 × 150 × 6 mm^3^ was reported. The designed antenna consists of a typical inverted-F antenna and an asymmetric T-shaped slot and can operate over 790–990 MHz and 1650–2920 MHz bands. In Ref.^16^, a 3D loop antenna was presented for multiband smartphone applications. It consists of a loop radiator and a large system ground plane and can operate over 0.8–1.1 GHz and 1.7–2.58 GHz bands. The antennas reported in Refs.^3,6,9,16^ can cover seven service bands (the GSM850, GSM900, DCS, PCS, UMTS, LTE2300, LTE2500), but WMT, WiFi, WiMAX, WLAN, and 5G bands are not covered. An octa-band antenna for LTE mobile phones was presented in Ref.^4^. The studied antenna consists of two monopole branches, two ground branches, and a matching circuit. Having a size of 79 × 142 × 7.5 mm^3^, it achieved dual impedance bands of 665–965 MHz and 1610–2800 MHz. In Ref.^7^, an octa-band monopole antenna was presented for WWAN/LTE applications. The designed antenna is comprised of a folded metal plate with a lumped-element high-pass matching circuit and can achieve dual operating bands of 0.69–0.98 GHz and 1.63–2.74 GHz. For mobile phones in Ref.^8^, an octa-band antenna was studied. The presented design consists of a coupled line, a monopole branch, and a ground branch. Using the λ/4-, λ/2-, and 3λ/4-resonant modes, the reported antenna can achieve dual operating bands of 0.67–1.02 GHz and 1.65–2.92 GHz. For LTE/WWAN operation in Ref.^12^, a linear open slot antenna was reported. With an overall dimension of 75 130 × 6 mm^3^, the studied antenna achieved dual operating bands of 698–960 MHz and 1710–2690 MHz. In Ref.^13^, an octa-band antenna that operates over 0.675–1.05 GHz and 1.6–2.8 GHz bands was reported. It is comprised of two branches and a matching circuit and possesses an overall size of 70 × 127 × 6 mm^3^. A wideband monopole antenna for an octa-band mobile phone application was reported in Ref.^14^. The studied antenna consists of a T-shaped driven strip and a dual-branch parasitic ground strip and achieved dual impedance bands 690–970 MHz and 1680–2740 MHz. A compact multi-band monopole antenna for WWAN/LTE mobile phones was proposed in Ref.^15^. The antenna is comprised of two branches and a matching circuit. With an overall volumetric size of 70 × 128 × 4.8 mm^3^, the studied antenna can work at 0.697–1.01 GHz and 1.59–3.25 GHz bands. The antennas reported in Refs.^4,7,8,12–15^ can cover eight service bands including LTE700, GSM850, GSM900, DCS, PCS, UMTS, LTE2300, and LTE2500 but they have failed to operate at WMT, WiFi, WiMAX, WLAN, and 5G bands. In Ref.^10^, a nine-band hybrid loop/open-slot antenna was presented for the LTE operation. The antenna is consisting of a capacitively-fed loop antenna and a ground strip and can attain triple working bands of 698–960 MHz, 1710–2690 MHz, and 3400–3800 MHz and can cover the above-mentioned bands and LTE3400. In Ref.^11^, a narrow-frame antenna was presented mobile phone application. The design is comprised of a monopole with four branches and a dual-branch ground strip. With an overall size of 75 × 140 × 5.8 mm^3^, the studied antenna attained five impedance bands of 681–991 MHz, 1626–2706 MHz, 3300–3813 MHz, 5136–5379 MHz, and 5622–6000 MHz. The designed antenna can operate over LTE700, GSM850, GSM900, DCS, PCS, UMTS, LTE2300, LTE2500, LTE3400 for 2G/3G/4G, 3.5 GHz for WiMAX and 5.2, 5.8 GHz bands for WLAN service bands, but the WMT, WiFi5.5, and 5G bands are not covered. In Ref.^5^, a 13-band antenna was presented for 4G/5G/WLAN applications. The designed antenna is comprised of four ground branches and a coupled line. With an overall size of 70 × 140 × 7 mm^3^, it achieved four operating bands and can cover the LTE700, GSM850, GSM900, DCS, PCS, UMTS, LTE2300, LTE2500, LTE3400, 2.4, 5.2, 5.8 GHz bands for WLAN, the 3.5 GHz and 4.8 GHz bands for 5G system but do not cover the WMT, WiFi and WiMAX band. Though many of the reported antennas successfully achieved multiple operating bands covering 7/8/9/11/13 service bands, they have the demerits of complex design, large volumetric size, and 3D profile. Moreover, most of them use lumped elements as a matching circuit which increases the fabrication cost and complexity. To the best of our knowledge, very few antennas are reported that can cover the 16 popular service bands including the LTE850, GSM850, GSM900, UMTS, LTE2190/2000S band, LTE3500, LTE 3700, and LTE5537.5 bands for 4G/3G/2G systems, 1427–1432 MHz band for WMT, 900 MHz and 5.8 GHz bands for WiFi, 3.5 GHz, and 5.5 GHz bands for WiMAX, 5.8 GHz band for WLAN and the 3.5 GHz and 4.8 GHz bands for 5G system.

In this paper, an octa-band printed monopole antenna is presented for 2G, 3G, 4G, WiFi, WiMAX, WLAN, and 5G communication applications. The studied antenna comprises an inverted F-shaped monopole radiator and an F-shaped ground strip. To excite multiple resonant modes, two rotated L-shaped strips are respectively added to the radiator and ground strip and thus the antenna can achieve eight operating bands of 824–905 MHz, 1.436–1.584 GHz, 1.994–2.318 GHz, 2.998–3.324 GHz, 3.574–3.72 GHz, 4.123–4.363 GHz, 4.562–5.024 GHz and 5.404–6.00 GHz. The proposed antenna not only has a simple planar profile but also be able to cover sixteen narrow service bands. Besides, one prime advantage of the studied antenna is that it does not use any lumped element to match the antenna and kept ample of free spaces for other circuitries.

## Antenna design and analysis

**Figure 1:**
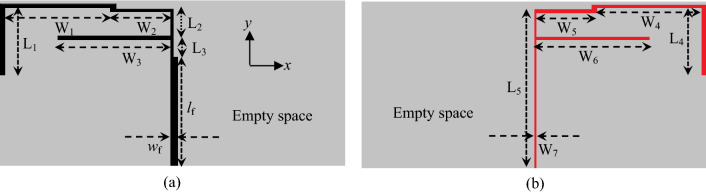
The geometry of the proposed antenna (**a**) Top view, and (**b**) bottom view.

The layout of the proposed octa-band antenna is shown in Fig. 1. It is comprised of an inverted F-shaped monopole radiator and a ground strip. As shown in Fig. 1a an unequal arm inverted-F-shaped patch with a rotated L-shaped branch is printed on the front side of 1.6 mm-thick FR4 substrate material with dielectric constant 4.3 and is fed by a feed line of 50 Ω characteristics impedance. The F-shaped ground with a rotated L-shaped branch is printed on the other side of the substrate as displayed in Fig. 1b. The width and length of the feedline are respectively *w*_f_ and *l*_f_. The monopole radiator with the rotated L-shaped strip is connected to the feed line through a microstrip line while the ground strip is terminated with another microstrip line. To achieve good impedance matching between the radiator and ground strip, the width and length of each branch of the patch and ground are optimized by CST simulation software. The optimized design parameters of the studied antenna are as follows: *L*_1_ = 41.6 mm, *L*_2_ = 17.38 mm, *L*_3_ = 11.80 mm, *L*_4_ = 41.8 mm, *L*_5_ = 93.54 mm, *L*_f_ = 64.36 mm, *W*_f_ = 2.36 mm, *W*_1_ = 62.62 mm, *W*_2_ = 36.23 mm, *W*_3_ = 67.35 mm, *W*_4_ = 62.56 mm, *W*_5_ = 36.29 mm, *W*_6_ = 67.35 mm, and *W*_7_ = 0.75 mm.

### Design evolution

**Figure 2:**
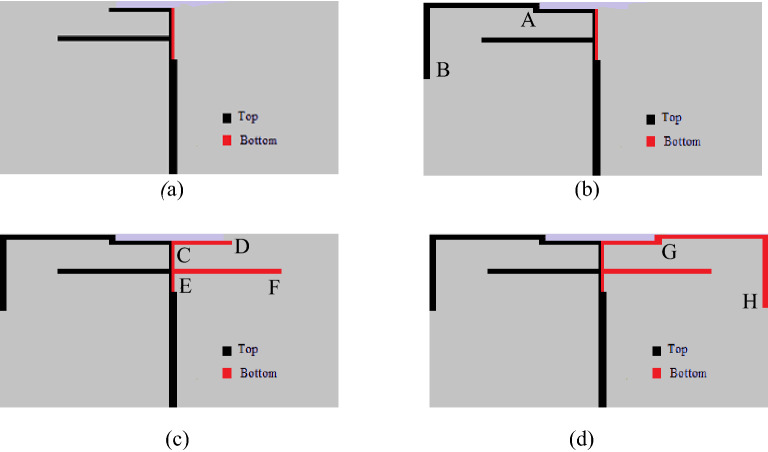
Four evolution stages of the antenna design (**a**) Ant-1, (**b**) Ant-2, (**c**) Ant-3, (**d**) the proposed antenna.

**Figure 3:**
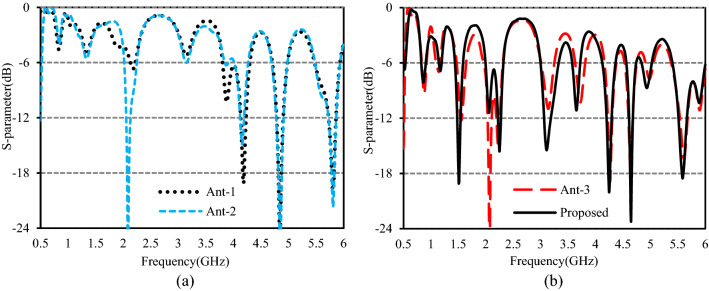
Simulated S-parameters for (**a**) Ant-1, and Ant-2, (**b**) Ant-3 and the proposed antenna.

To comprehend the working principle of the studied antenna, a comparison of the simulated S-parameters of four different evolution stages is presented in Fig. 3. The four evolution stages of the presented antenna are displayed in Fig. 2. In Fig. 2a Ant-1 is shown which consists of a microstrip line fed inverted F-shaped monopole radiator and a vertical strip line in the ground. A rotated L-shaped branch AB is added to the monopole of Ant-1 to form the Ant-2 is shown in Fig. 2b. As in Fig. 2c, Ant-3 is formed by adding two horizontal strip lines CD and EF with the vertical line of the ground to form an F-shaped ground plane. Finally, another rotated L-shaped branch GH is merged with horizontal line CD to form the proposed antenna as shown in Fig. 2d. The simulated S-parameters presented in Fig. 3a show that the Ant-1 achieved the operating bands of 2.134–2.200 GHz, 3.806–4.295 GHz, 4.708–4.997 GHz, and 5.472–5.934 GHz. Though it can cover WCDMS/IMT-2000/UMTS, 2000 S-band, LTE2190, LTE5537.5, WiMAX 5.5, WiFi 5.8, WLAN 5.8, it has insufficient bandwidth to cover GSM850, GSM900, UMTS, WiFi 860, LTE 850, LTE880, LTE3400, the 3.5 GHz for 5G, and WiMAX 3.5 GHz bands.

The S-parameter of Ant-2 in Fig. 2a shows, the lower resonance at 2.178 GHz moves down to 2.084 GHz with an improved S_11_ value of − 28.2 dB. The shifting of this resonance helps to improve the bandwidth of the lower band. However, the operating bands are still unsatisfactory. When the horizontal strip line CD and EF are added to the vertical ground strip (Ant-3), the impedance matching is remarkably improved as shown in Fig. 3b. Figure 3b shows that the addition of branches CD and EF to the vertical ground strip of Ant-2 excites six additional resonances at around 874 MHz, 1.144 GH.140 GHz, 3.723 GHz, and 4.983 GHz that can help to cover the GSM850, UMTS, GSM900, WiFi 860 GHz, LTE850, LTE880, LTE3500, LTE3700, 3.5 GHz for 5G sub-6 GHz band, WiMAX 3.5 GHz band. When the rotated L-shaped branch GH is added to the Ant-3(the proposed antenna), the resonances at 1.144 GHz, 3.140 GHz, and 4.983 GHz shifted downward and upward resulting in enhancement of operating bandwidth that helps to cover the entire 4.8 GHz (4.6–5.0 GHz) band for 5G sub-6 GHz communication system.

### Current distributions

**Figure 4:**
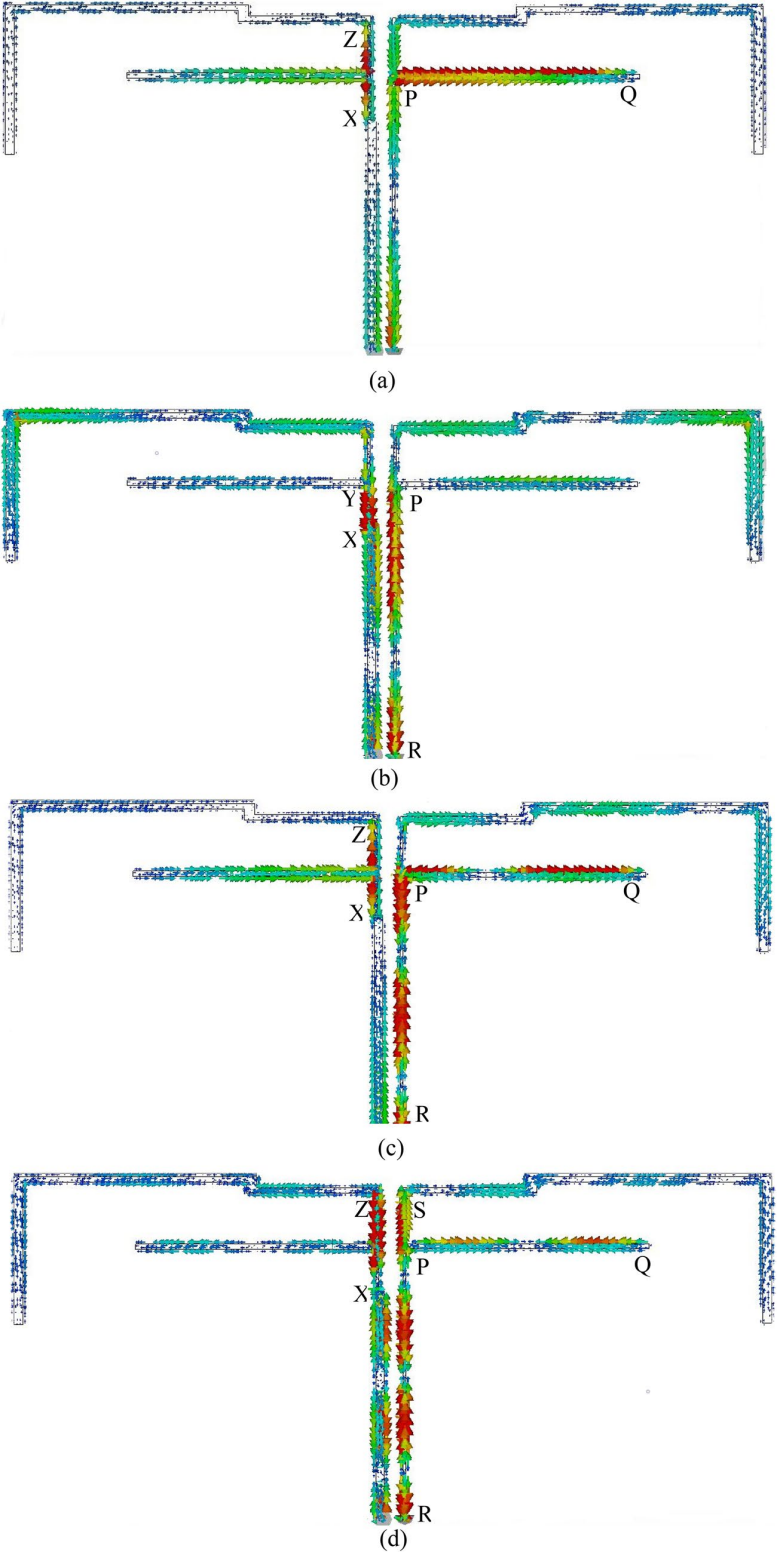

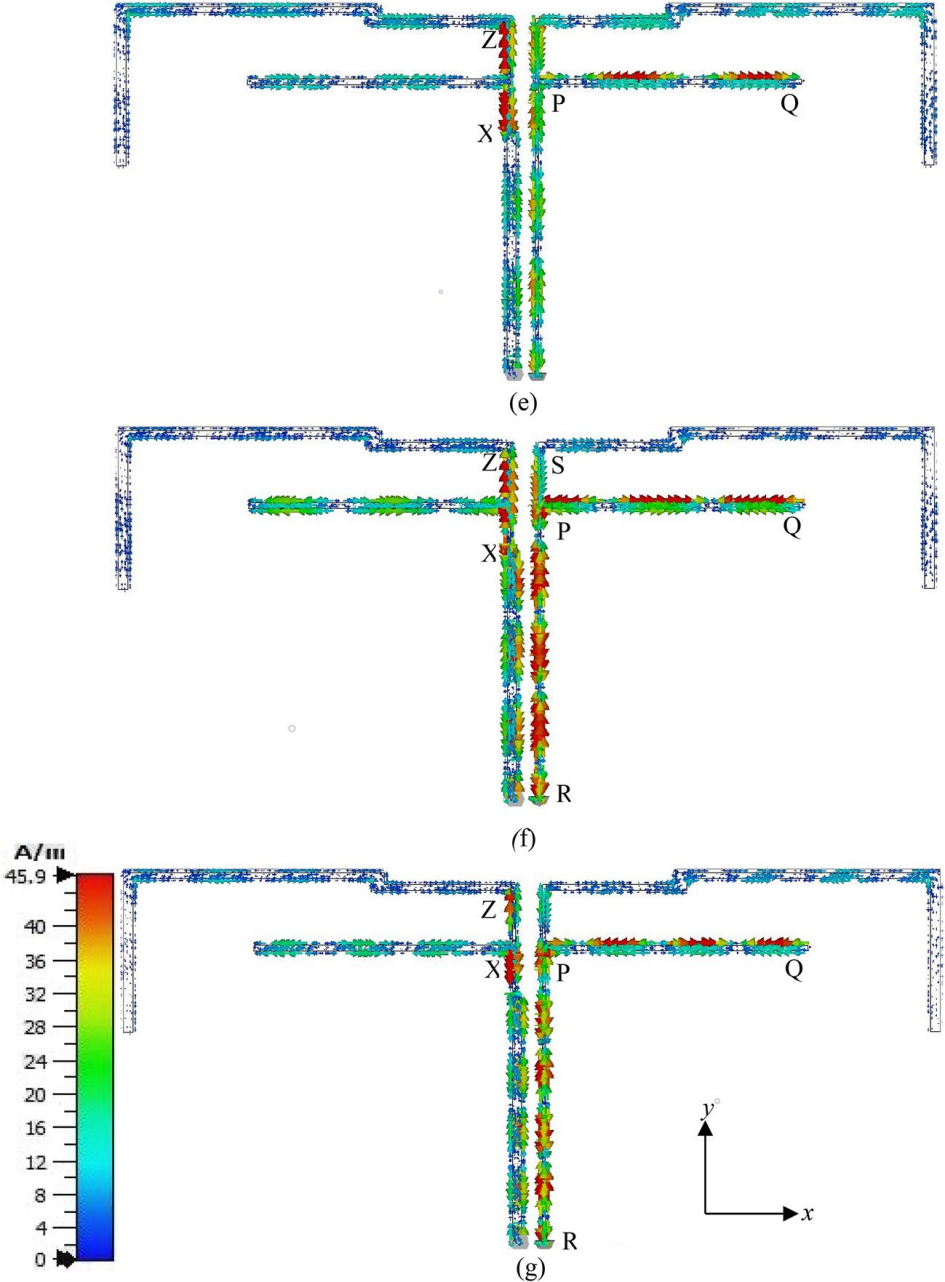
Surface current distributions at (**a**) 863 MHz, (**b**) 1.51 GHz, (**c**) 2.25 GHz, (**d**) 3.11 GHz, (**e**) 3.65 GHz, (**f**) 4.25 GHz, (**g**) 5.59 GHz.

**Figure 5:**
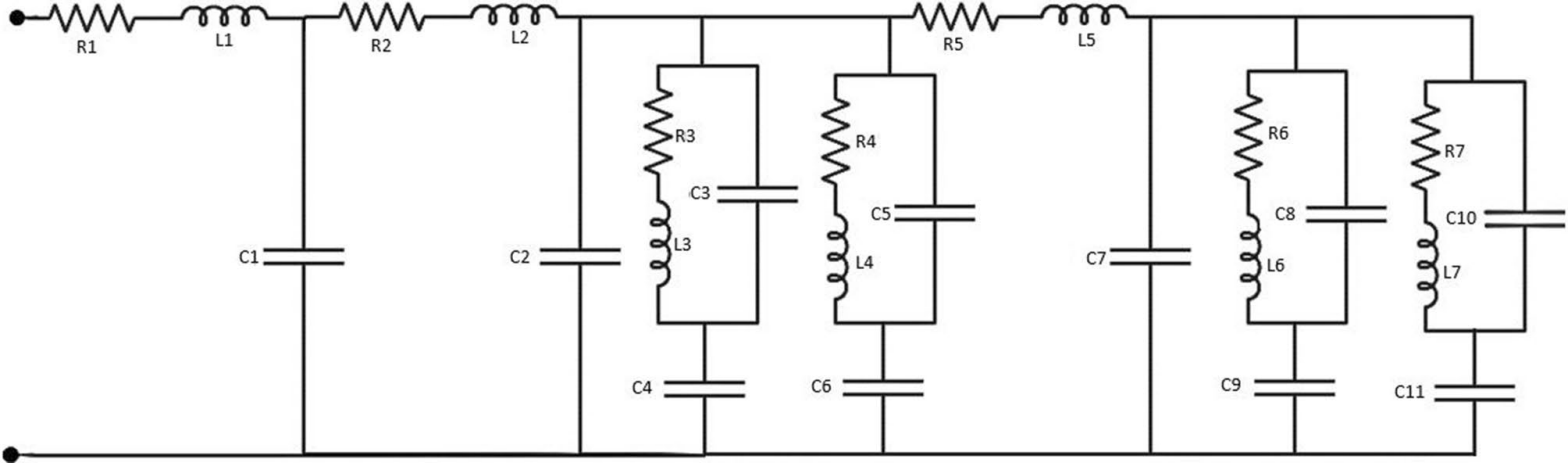
Equivalent circuit of the proposed antenna.

To realize the working principle of the studied antenna, the current distributions at resonant frequencies of 863 MHz, 1.51 GHz, 2.25 GHz, 3.11 GHz, 3.65 GHz, 4.25 GHz, and 5.59 GHz are shown in Fig. 4a–g. For clarity, the current distribution on the monopole radiator and ground strip is displayed side by side. At 863 MHz, the current is strong at branch PQ of the ground strip and section XZ of the inverted F-shaped radiator. As Fig. 4a shows, the current is strong at point P and it is weak at point Q. This means that the resonance at 863 MHz is associated with 0.25-λ mode of the ground branch PQ which can be tuned with the help of XZ which is supported by Fig. 3b. At 1.51 GHz as shown in Fig. 4b, the current is more intense at section RP of the ground strong at both points R and P. Consequently, resonance at 1.51 GHz is associated with 1.5 × 0. 25-λ mode of section RP which can be adjusted with the help of section XY of the vertical monopole. As in Fig. 4c, at the 3rd resonance frequency of 2.25 GHz, the current is highly concentrated at section RPQ of the ground strip and XZ section of the radiator. The current is strong at points R and P and it is weak at point Q. Three nulls also have been observed at section RPQ which indicated that this resonance is related to higher-order modes of section RPQ. At 3.11 GHz as displayed in Fig. 4d, the current is more intense at the sections RPS and PQ of the ground strip and XZ section of the monopole radiator. Thus, the resonance at 3.11 GHz is generated by sections RPS and PQ section of the ground strip with the help of section XZ and it is associated with the 1-λ mode of section RPS of length *l*_5_. As displayed in Fig. 4e, the current concentration at section XZ of the monopole strip and PQ of the ground strip is higher. Thus, the resonance at 3.65 Hz is mainly generated by section XZ of the monopole radiator and tuned to lower frequency by section PQ which is demonstrated in Fig. 3. The current is strong at both points X and Z and weak at points P and Q and there are nulls in between them. Therefore, the antenna works at around 0.5-λ mode of section XZ and around 0.75-λ mode at the section of PQ. At 4.25 GHz as shown in Fig. 4f, the current at sections XZ of the monopole and RPS and PQ of the ground strip is stronger. The current at points X, Z, R, P, Q are stronger and weak at S, and numbers of nulls have been observed in section RPS. Ignoring the radiation from the microstrip line, it can be said that at 4.25 GHz the antenna excites at 0.5-λ mode of section XZ and higher modes of section RPS and PQ. As displayed in Fig. 4g the current concentration at 5.59 GHz is higher at sections XZ of the monopole and RPQ of the ground strip. Therefore, the resonance at this frequency is generated by section XZ and shifted to the lower band by section RPQ as shown in Fig. 3. The current is strong at points X, Z, R, P, and Q. A null has been observed in between points X and Z and numbers of nulls have been observed in between points R and Q, thus it can be commented that 0.5-λ mode of section XZ and higher-order modes of section RPQ are excited at 5.59 GHz. As conclusions, using an inverted F-shaped monopole radiator and the 0.25-λ, 0.5-λ, and 1-λ modes of vertical and lower arms of the F-shaped ground strip, the studied antenna achieved desired multiple operating bands which was reported in Refs.^8,11–13^. Moreover, the equivalent circuit of the proposed antenna is designed based on transmission line theorem, where each metal part has the effect of inductance and gap between two conductor acts as the capacitor, presented in Fig. 5.

### Parametric studies

**Figure 6:**
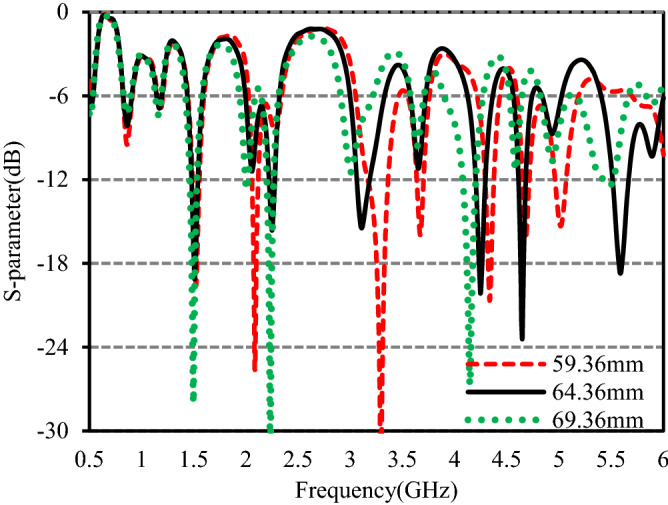
S-parameters for different values of *l*_f_.

**Figure 7:**
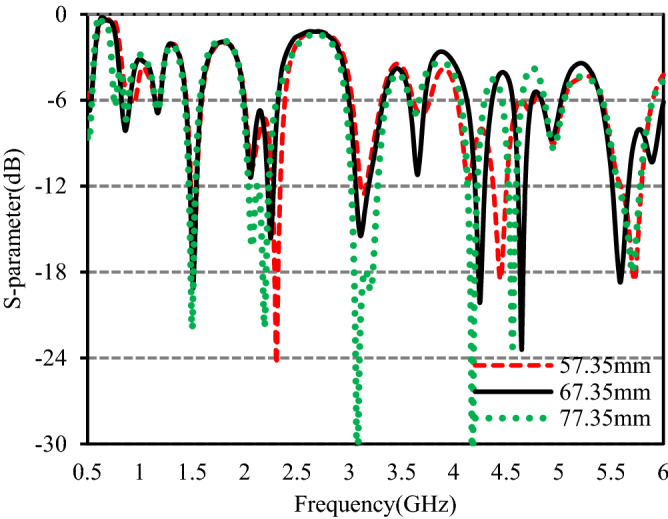
S-parameters for different values of *W*_3_.

**Figure 8:**
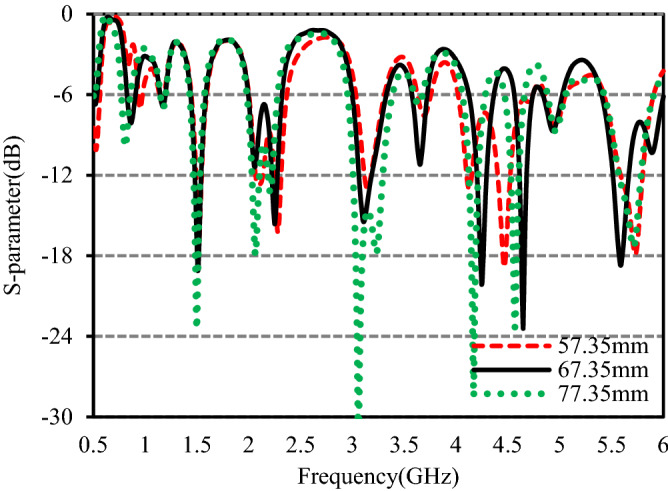
S-parameters for different values of *W*_6_.

To better comprehend the behavior of the anticipated antenna, the effects of *l*_f_, *W*_3_, and *W*_6_ are analyzed and presented in Figs. 6, 7 and 8. The simulated S-parameters with different values of *l*_f_ that are related to the length of feedline of the monopole are shown in Fig. 6. When the value of *l*_f_ varies from 59.36 to 69.36 mm, at the 1st, 2nd, and 3rd bands, there is little change in the resonant frequencies and operating bandwidth. At the 4th band, the bandwidth is decreased, and resonance frequency moves towards the lower band with the worst matching. In the 5th band, the matching deteriorated and resonance frequency moves from 3.29 to 2.98 GHz with the decrement of bandwidth. In the 6th band, the bandwidth slightly decreases and resonance frequency moves downward with lower S_11_. At the7th band, bandwidth is increased and the resonance frequency moves from 4.33 to 4.15 GHz. At the 8th band, bandwidth is decreased and the resonance frequency down to 4.58 GHz from 4.67 GHz. The resonance frequency of the 9th band shifts towards the lower band and the bandwidth is decreased. At the 10th band, the matching is improved and resonance moves towards the lower operating frequencies. Trading off the bandwidth and the matching, *l*_f_ = 64.36 mm is taken as the optimized value.

Figure 7 shows the simulated S-parameters for different values of *W*_3_, the length lower branch of the inverted F-shaped monopole radiator. It can be observed from the plot that *W*_3_ has no significant effect on the 2nd, 3rd, and 9th operating bands. The resonance frequency of the 1st band moves towards the lower band as the value of *W*_3_ increases and the matching becomes worse for values higher and lower than the optimized one. The bandwidth of the 4th band at 2.25 GHz decreases with increasing *W*_3_ whereas the bandwidth of the 5th band at 3.11 GHz increases with increasing *W*_3_. For the values lower and higher than the optimized one, the matching at the 6th band at 3.65 GHz becomes worse. The bandwidth of the 7th band at 4.25 GHz increases with decreasing *W*_3_ despite of poor S_11_ value. When the value of *W*_3_ varies from 57.35 to 77.35 mm, the resonance frequency of the 8th band is shifted from 4.44 to 4.56 GHz and the bandwidth is decreased. Great dependence of the 10th band at 5.59 GHz on *W*_3_ has been observed in the plot.

Trading off the bandwidth and the impedance matching, a value of 67.35 mm is taken as the final value to fabricate the studied antenna.

The S-parameters with different values of *W*_6_, length of the lower branch of the F-shaped ground strip are shown in Fig. 8. The variation of *W*_6_ has no significant effect on the 2nd, 3rd and 9th bands, while the other operating bands are affected by *W*_6_. The bandwidth of the 1st band at 863 MHz is increased with increasing *W*_6_ and its resonant frequency moves towards the lower operating band. When the value of *W*_6_ varies from 57.35 to 77.35 mm, the bandwidth of the 4th band at 2.25 GHz is decreased whereas the bandwidth of the 5th band at 3.11 GHz is increased due to improved matching. The matching at the 6th band becomes poorer as the value of *W*_6_ is lower or higher than the optimized one. The matching at the 7th and 8th bands is improved with increasing *W*_6_ along with shifting of resonance frequencies from the desired one. The bandwidth 10th band becomes smaller and the matching is poorer as the value of *W*_6_ is lower or higher than the optimized value. To get the desired operating bands with improved matching the final value of *W*_6_ is taken as 67.35 mm.

## Experimental results and discussions

**Figure 9:**
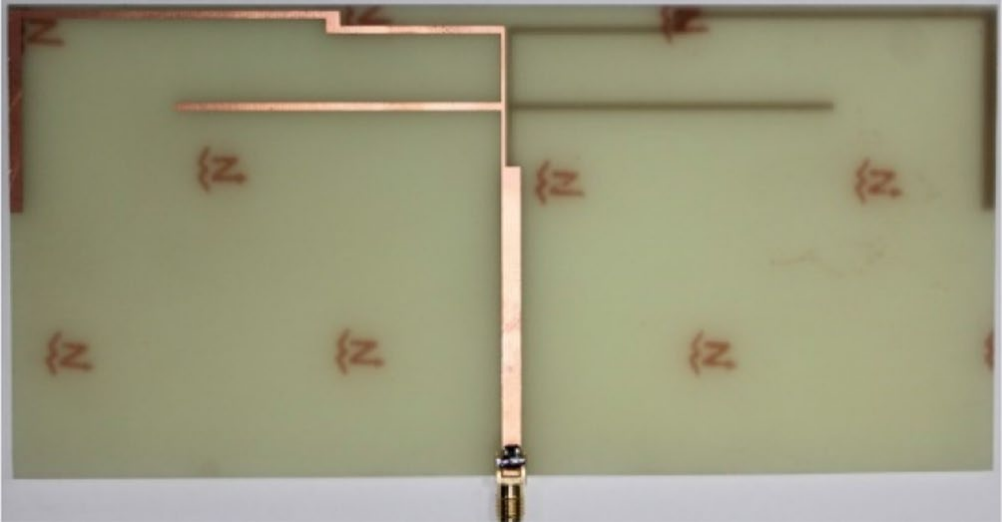
Photograph of the fabricated antenna.

**Figure 10:**
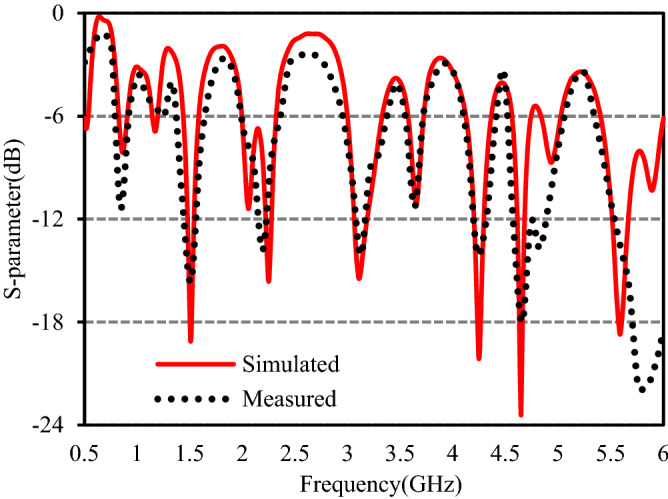
Simulated and measured S-parameters.

The studied antenna has been fabricated with optimized parameters and is shown in Fig. 9. The input impedance characteristic of the presented antenna is measured with the help of the Agilent N5227A network analyzer and the S-parameter responses are presented in Fig. 10. It is evident from the plot that the measured − 6 dB operating bands of the studied antenna are 789–921 MHz (132 MHz), 1367–1651 MHz (284 MHz), 1995–2360 MHz (365 MHz), 2968–3374 MHz (406 MHz), 3546–3707 MHz (161 MHz), 4091–4405 MHz (314 MHz), 4519–5062 MHz (543 MHz) and 5355–6000 MHz (645 MHz). Hence, the prototype antenna achieved eight operating bands and can cover sixteen well-established service bands. The measured result agrees well with the simulated one. The slight discrepancy is caused by fabrication errors.

**Figure 11:**
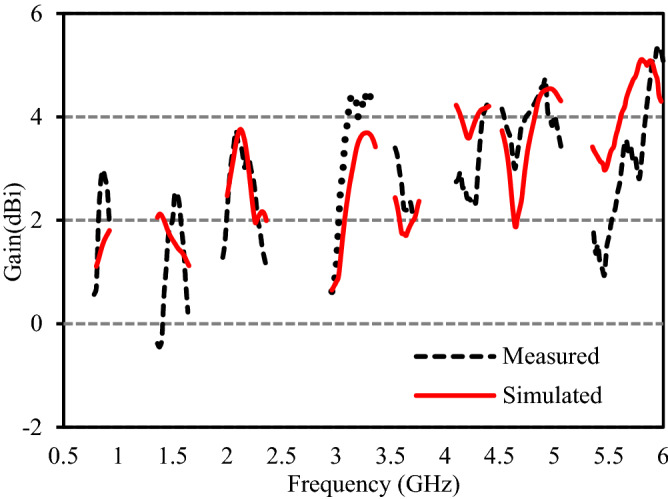
Measured and simulated peak gain.

**Figure 12:**
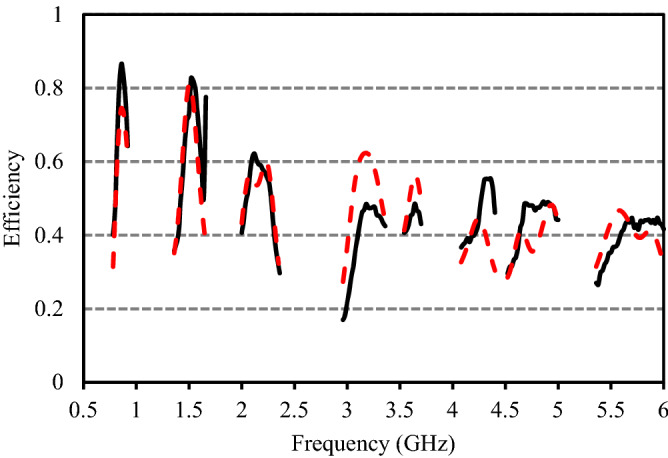
Measured and simulated radiation efficiency.

The radiation characteristics of the studied multi-band antenna are measured in an anechoic chamber using near field antenna measurement system StarLab from MVG. The gain and efficiency of the proposed antenna are respectively shown in Figs. 11 and 12. It is observed from Fig. 10 that at the eight operating bands, the average measured gains are 2.04 dBi, 1.19 dBi, 2.57dBi, 3.48 dBi, 2.60 dBi, 3.05 dBi, 4.94 dBi, and 3.11 dBi, respectively. At the first, second, third, fourth, fifth, sixth, seventh, and eighth impedance bands, the maximum measured efficiency of the presented antenna are respectively 86.6%, 82.7%, 62.2%, 48.5%, 48.6%, 55.4%, 49.1%, and 44.8% and is acceptable for cellular communications^3–5,9,19–21^. The efficiency of the proposed antenna in the 4th, 5th, 7th, and 8th bands is slightly less than 50% which is due to the excitation of surface waves. In the proposed antenna, the surface waves spread out around the excitation point and are reflected when they meet the ground plane and at the dielectric-to-air boundary. The fields remain trapped within the substrate and absorb part of the energy of the signal resulting in the decrement of efficiency^22^. Moreover, the low efficiency of the antenna is due to the higher loss of standard FR4 substrate used to fabricate the antenna. When a substrate with low dielectric permittivity is used, less energy is coupled into the surface waves, thus reduces the magnitude of the surface waves which in turn increases the efficiency. Moreover, the efficiency of the proposed antenna can also be increased using reflecting surfaces, active component, EBG structure, slot matching, and substrate with the low loss^23^.

**Figure 13:**
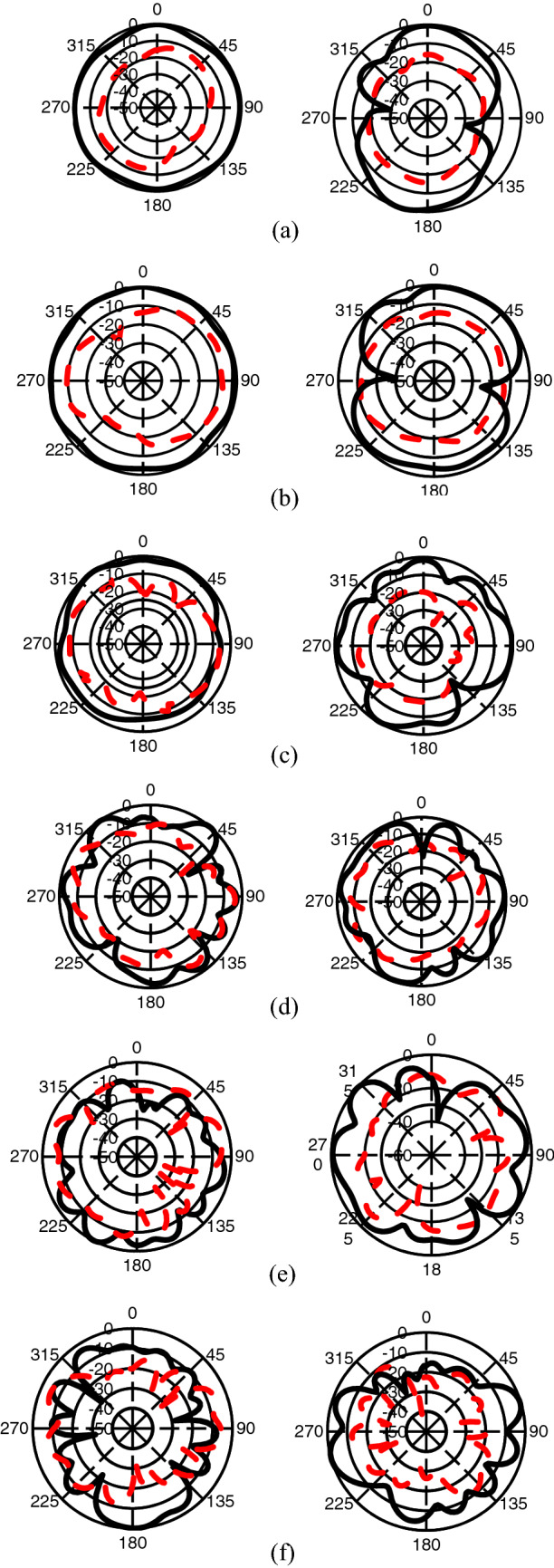
Measured normalized *E*-(left) and *H*-(right) field patterns at (**a**) 863 MHz, (**b**) 1.51 GHz, (**c**) 2.25 GHz, (**d**) 3.11 GHz, (**e**) 4.25 GHz, (**f**) 5.59 GHz.

The measured normalized radiation patterns of the studied antenna at 863 MHz, 1.51 GHz, 2.25 GHz, 3.11 GHz, 4.25 GHz, and 5.59 GHz are depicted in Fig. 13. In the plot, the black solid lines represent the co-polarized component (*E*_φ_) while the red dashed lines represent the cross-polarized component (*E*_θ_). At 863 MHz, 1.51 GHz, and 2.25 GHz shown in Fig. 13a–c respectively, the *E*-field pattern is almost omnidirectional and the *H*-field pattern is dipole-like with good omnidirectional radiation which indicates that at lower frequencies, the studied antenna exhibits stable radiation characteristics. This characteristic is similar to the observation in many wideband/multiband slot antennas reported in Refs.^24–26^. At higher frequencies of 3.11 GHz, 4.25 GHz, and 5.59 GHz, respectively displayed in Fig. 13d–f, comparatively large variations both in the *E*-field and *H*-field radiation patterns are observed. These rapid variations in the radiation characteristics are mainly due to the nulls of the surface current distribution on the ground strip. In both *E*- and *H*-plane, the cross-polarized level, and distortion are respectively higher at low and frequencies which may be due to the excitation of surface/leaky waves. Surface waves propagate until they reach an edge of the substrate. When surface waves reach these boundaries, they are reflected and diffracted by the edges. The diffracted waves provide an additional contribution to radiation, degrading the radiation patterns by producing distortions, and cross-polarization levels^27^. This higher level of cross-polarized radiation may also be due to the undesirable higher-order radiating modes that affect the far-field performance of the anticipated antenna. Moreover, as the studied antenna is prototyped on substrate material with higher thicknesses and large dielectric constant, this cross-polarized radiation becomes significantly prominent near the broadside direction which reflects the finding reported in Refs.^14–16,28^. This higher cross-polarized level and distortions can be suppressed using modified feeding arrangement, exploration of defected patch surface, use of the shorted patch, use of microwave absorbers, and metamaterials.

To accent, the advantages, the size and performance of the studied antenna are compared with recently reported multi-band antennas and are listed in Table 1. Obviously, the overall size of the proposed antenna is compact than those reported in Refs.^3–16^ while achieving more operating bands sufficient to cover 16 popular service bands. Though some of the reported antennas achieved higher gain and efficiency, they possess complex structure, 3D profile, and use lumped element matching circuit. Moreover, none of the antennas reported in Refs.^3–16^ cover as many service bands as the proposed antenna does. As the studied antenna possesses a planar profile and requires no vertical space/large system ground plane or lumped elements, the prototyping of the presented antenna is simpler than those in Ref.^3–16^. Therefore, the advantageous features of the proposed antenna such as small volumetric size, higher operating bands, and ease of fabrication make it suitable for sixteen narrow service bands in 2G, 3G, 4G, WiFi, WiMAX, WLAN, and newly deployed 5G sub-6 GHz communication systems.

**Table 1:** Comparative study of the few existing multi-band antennas with the proposed antenna.

References	Overall size (mm^3^)	Volume (mm^3^)	Operating bands (MHz)	Used lumped elements	Peak gain (dBi)	Max. efficiency (%)	Substrate material
^3^	150.8 × 200.8 × 7	211,964	746–960	Yes	NA	83	FR4
			1710–2690				
^4^	79 × 142 × 7.5	84,135	665–965	Yes	NA	69	FR4
			1610–2800			81	
^5^	70 × 140 × 7	68,600	698–960	Yes	3	69.9	FR4
			1710–2690		3.9	69.4	
			3300–3800		3.3	59.4	
			4600–5850		4.5	67.7	
^6^	76 × 150 × 6	68,400	824–960	Yes	2.1	48	FR4
			1710–2690		5.1	76	
^7^	80 × 140 × 5.8	64,960	690–980	Yes	3.0	94.5	FR4
			1630–2740		5.6	73.2	
^8^	80 × 140 × 5.8	64,960	0.67–1.02	No	3.37	82	FR4
			1.65–2.92		4.48	97.2	
^9^	70 × 150 × 6	63,000	790–990	Yes	NA	72.6	FR4
			1650–2920			84.4	
^10^	75 × 140 × 5.8	60,900	698–960	Yes	NA	65	FR4
			1710–2690			92	
			3400–3800				
^11^	75 × 140 × 5.8	60,900	681–991	No	5.93	81.1	Rogers
			1626–2706				
			3300–3813				
			5136–5379				
			5622–6000				
^12^	75 × 130 × 6	58,500	698–960	Yes	NA	63	FR4
			1710–2690			95	
^13^	70 × 127 × 6	53,340	675–1050	Yes	0.1	78.7	FR4
			1600–2800		3.6	81	
^14^	70 × 128 × 5.8	51,968	690–970	No	3.24	86.9	FR4
			1680–2740		4.02	82.9	
^15^	70 × 128 × 4.8	43,008	697–1010	Yes	2.52	83.6	FR4
			1590–3250		4.7	89.9	
^16^	60 × 120 × 5	36,000	800–1100	Yes	2.4	76	Rogers
			1700–2580		4.5	84	
This work	97.6 × 205 × 1.6	32,013	789–921	No	2.98	86.6	FR4
			1367–1651		2.54	82.7	
			1995–2360		3.74	62.2	
			2968–3374		4.44	48.5	
			3546–3707		3.40	48.6	
			4091–4405		4.29	55.4	
			4519–5062		6.32	49.1	
			5355–6000		5.32	44.8	

## Conclusion

To fulfill the demand of wireless communication towards next-generation and to operate over multiple frequency bands, a planar monopole antenna is presented for portable communication devices. The anticipated antenna consists of an inverted F-shaped monopole patch and an F-shaped ground plane. The multi-resonant modes can easily be excited and controlled by different branches of the patch and vertical and lower branches of the ground strip. For S_11_ ≤ − 6 dB, the fabricated antenna achieved eight operating bands and can cover sixteen well-established service bands. The measured peak gains at the eight operating bands are 2.98, 2.54, 3.74, 4.44, 3.40, 4.29, 6.32, and 5.32 dBi respectively. The designed octa-band antenna also achieved satisfactory radiation efficiency and exhibits nearly omnidirectional radiation patterns which make it very suitable for being used in portable communication devices such as tablets, laptops, etc. The main advantage of the anticipated antenna is to cover sixteen narrow service bands in the 2G, 3G, 3G, 5G sub-6 GHz, WiFi, WiMAX, and WLAN communication systems without any lumped elements and keeping sufficient empty spaces for other microwave circuitries.
